# A comparison of drone imagery and ground-based methods for estimating the extent of habitat destruction by lesser snow geese (*Anser caerulescens caerulescens*) in La Pérouse Bay

**DOI:** 10.1371/journal.pone.0217049

**Published:** 2019-08-09

**Authors:** Andrew F. Barnas, Brian J. Darby, Gregory S. Vandeberg, Robert F. Rockwell, Susan N. Ellis-Felege

**Affiliations:** 1 University of North Dakota, Department of Biology, Grand Forks, North Dakota, United States of America; 2 University of North Dakota, Department of Geography & Geographic Information Science, Grand Forks, North Dakota, United States of America; 3 American Museum of Natural History, Vertebrate Zoology, New York, New York, United States of America; Universitat Autonoma de Barcelona, SPAIN

## Abstract

Lesser snow goose (*Anser caerulescens caerulescens*) populations have dramatically altered vegetation communities through increased foraging pressure. In remote regions, regular habitat assessments are logistically challenging and time consuming. Drones are increasingly being used by ecologists to conduct habitat assessments, but reliance on georeferenced data as ground truth may not always be feasible. We estimated goose habitat degradation using photointerpretation of drone imagery and compared estimates to those made with ground-based linear transects. In July 2016, we surveyed five study plots in La Pérouse Bay, Manitoba, to evaluate the effectiveness of a fixed-wing drone with simple Red Green Blue (RGB) imagery for evaluating habitat degradation by snow geese. Ground-based land cover data was collected and grouped into barren, shrub, or non-shrub categories. We compared estimates between ground-based transects and those made from unsupervised classification of drone imagery collected at altitudes of 75, 100, and 120 m above ground level (ground sampling distances of 2.4, 3.2, and 3.8 cm respectively). We found large time savings during the data collection step of drone surveys, but these savings were ultimately lost during imagery processing. Based on photointerpretation, overall accuracy of drone imagery was generally high (88.8% to 92.0%) and Kappa coefficients were similar to previously published habitat assessments from drone imagery. Mixed model estimates indicated 75m drone imagery overestimated barren (F_2,182_ = 100.03, P < 0.0001) and shrub classes (F_2,182_ = 160.16, P < 0.0001) compared to ground estimates. Inconspicuous graminoid and forb species (non-shrubs) were difficult to detect from drone imagery and were underestimated compared to ground-based transects (F_2,182_ = 843.77, P < 0.0001). Our findings corroborate previous findings, and that simple RGB imagery is useful for evaluating broad scale goose damage, and may play an important role in measuring habitat destruction by geese and other agents of environmental change.

## Introduction

Light goose populations (lesser snow *Anser caerulescens caerulescens*, greater snow *A*. *c*. *atlanticus*, and Ross’s geese *A*. *rossii*) have grown rapidly since the 1960’s, predominately as a result of modernized agricultural practices in the southern extent of their ranges [1–3]. In their northern staging and summer breeding areas, growing numbers of light geese have dramatically altered vegetation communities through increased foraging pressure, resulting in a loss of above ground primary productivity [4, 5]. These impacts are especially well documented in colonies of lesser snow geese (hereafter snow geese), which have been formally designated as an overabundant species in Canada [1]. While snow goose foraging has direct impacts on vegetation communities, the indirect effects of this biomass loss have resulted in apparent trophic cascades in Canadian Arctic ecosystems with important consequences for sympatric species [5, 6]. Previous studies have linked growing snow goose colonies with decreased song bird nest occurrence [7, 8], reduced small mammal abundance [9] and reduced invertebrate community species richness [10, 11].

Continued monitoring and assessments of snow goose habitat damage is critical to management efforts to better predict the outcome of continued population growth, along with forecasting the effects of recently founded satellite colonies in new areas [12, 13]. Assessing plant-goose interactions is typically done using ground-based sampling designs (linear transects, quadrat sampling etc.), which offer high resolution data but are time consuming and logistically challenging in the remote regions where geese stage and breed [14, 15]. Further, in heterogeneous or highly degraded landscapes these logistically limited sampling methods may not adequately capture spatial variation in vegetation communities. As a result, local ecosystem processes may be poorly delineated, leading to weak inferences on regional patterns and trends. Remote sensing technologies such as satellites can offer opportunities to create broad regional indices such as the Normalized Difference Vegetation Index (NDVI) to quantify vegetation cover and have been used to study the relationships between geese and their forage plants [16–18]. These methods offer wide spatial coverage, but miss out on fine scale data that can be collected on the ground such as species assemblages or plant demographic information. Fortunately, satellite imagery resolution is continually improving. For example WorldView-03 (Satellite Imaging Corporation, Houston, Texas) offers panchromatic imagery at 0.31m/pixel and has been used for ecological research [19], but this imagery can be expensive and prone to interpretation errors [20]. More importantly, the quality of satellite imagery is dictated by prevailing atmospheric conditions such as cloud cover surrounding study sites, potentially limiting the repeatability of image acquisition and appropriate timing to address rapid landscape changes [21].

One solution to the problem of sampling scale and repeatability is the advent of drone [22] technology for ecological research [23, 24]. Drones are increasingly being used by ecologists to address questions involving vegetation communities and habitat assessments [25–27]. These platforms are able to rapidly collect high resolution imagery that can be easily archived for future analyses, and flight paths are highly repeatable over areas of interest which allows users to conduct repeated surveys with minimal variation. While recognizing that drone operations are still limited by environmental conditions (precipitation, high winds), smaller models can be rapidly deployed in the field when conditions become suitable, dampening logistic difficulties of organizing manned aircraft flights. This is especially relevant for research in polar regions with more persistent cloud cover, as drones are able to operate at low altitudes during cloudy conditions [28]. Clearly drones have great potential for monitoring the impact of snow geese and other agents of environmental change, which will ultimately help alleviate the high financial costs of research in the Arctic [29].

While studies in ecology featuring drones are on the rise, the many of these have been tested with small aircraft at restricted spatial scales [30–33]. Studies in wildlife featuring drones are currently restricted to flying within visual line-of-sight, but regulatory agencies are making strides towards relaxing these restrictions [34]. Indeed there are several examples of large-scale studies that have successfully used drones with beyond-visual-line-of-sight (BVLOS) flight plans [35–38] However, any aircraft models capable of very long distance surveys are not likely affordable to lone PIs or even collaborative research groups. For example, an increasingly popular long-range drone system, the ScanEagle (Insitu Inc., a subsidiary of The Boeing Company), costs an estimated $3.2 million US. Further, the operation of these aircraft requires a high degree of technical training, which is unlikely to be feasible for the average ecologist. Therefore, the future of large scale ecological research with drones is more likely to be outsourced to commercial operations, similar to satellite technology or even manned aircraft flights.

Some ecological studies have tested the capacity of long-range aircraft to acquire imagery at restricted spatial scales [39–41], setting the stage for routine acquisition and analysis of imagery collected BVLOS. The analysis of drone-based imagery collected by commercial operators may become analogous to methods used for satellite imagery, whereby the imagery is collected and ecological experts later interpret the imagery. This is not an unrealistic option for the future of assessing habitat degradation by snow geese at broad scales, given the high financial cost of field studies in the Arctic [29]. However before drones can be readily integrated into the toolkit of ecological researchers, validation studies are a necessary precursor to understand how interpretation of drone imagery by ecological experts compares to estimates made by field-based methods.

The objective of this study was to estimate the extent of habitat degradation in an area historically damaged by lesser snow geese using drone imagery. Specifically, we examine the composition of broad vegetation land cover classes using a standard field-based linear transect approach, and compare estimates to those made from the analysis of drone imagery via methods analogous to interpretation of commercially acquired imagery. We suspect that high resolution drone imagery will result in similar estimates of land cover estimates when compared to field-based sampling, which would therefore lead to similar inferences on biological processes. Further, we hypothesize that lower altitude flights with better image resolution will result in classifications of higher accuracy (based on photointerpretation) than flights at higher altitudes.

## Materials and methods

### Study area

**Fig 1:**
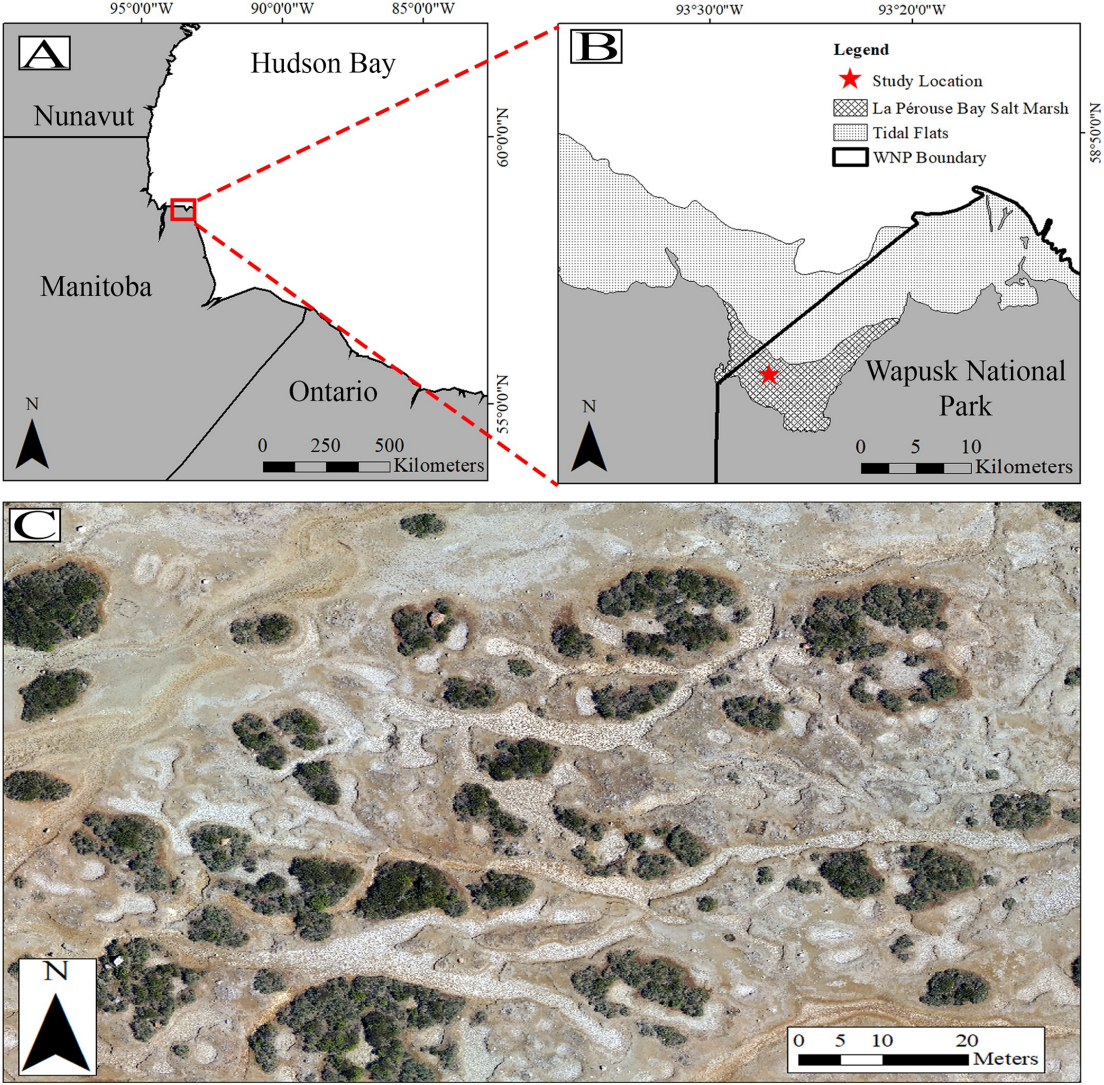
Map of study location. (A) Extent indicator of study location in northern Manitoba, (B) supratidal salt marsh study location within Wapusk National Park, (C) sample RGB photo of habitat surveyed by drone. Image acquired at 75 m above ground level.

This study was conducted at a long-term remote research camp within Wapusk National Park, Manitoba, Canada (Fig 1). This area is a coastal supratidal salt marsh, southwest of La Pérouse Bay along the western coast of Hudson Bay. The area is part of the Hudson Bay Lowlands physiographic region [42] and is characterized by a vegetation community predominately composed of dwarf shrub (*Salix sp*. *Betula glandulosa*, *Myrica gale*) and graminoid (e.g. *Puccinellia phryganodes*, *Festuca rubra*, *Triglochin sp*.) species. For a more detailed account of the region’s plant community and natural history see [5].

### Field derived estimates of land cover

We conducted ground-based linear transects within five rectangular study plots of varying size to evaluate vegetation. For further details on plot specifications see S1 Appendix. Each plot consisted of a grid system of adjacent 50x50m cells (92 cells, 23 ha total). Following methods established by previous habitat assessment studies in these plots, two linear transects were walked in each cell diagonally from northwest to southeast, and northeast to southwest corners [7, 43, 44]. Vegetation and landscape cover data along transects were collected following a modified step-point method whereby dominant composition was recorded underfoot approximately every meter [45, 46]. Vegetation was recorded to the genus or species level for target species of interest. For a complete list of classifications see S1 Appendix. Bare soils, waterways or ponds lacking vegetation, dried waterways or ponds, and dead willows were classified as barren. Dwarf shrub species in the genus *Salix*, *Betula*, and *Myrica* were classified as shrubs. All other plant species (predominately graminoids) were classified as non-shrubs. A single observer and a dedicated recorder conducted surveys from 12–19 July 2016.

### Drone data collection

We conducted drone flights using a Trimble UX5 (color: black, wingspan: 100 cm, weight: 2.5kg, cruise speed: 80 km/h), a fixed-wing rear-propelled aircraft powered by removable lithium polymer batteries (14.8V, 6000 mAh). The UX5 uses an elastic catapult launcher to initiate flights and engage the motor. We programmed the UX5 to follow a pre-defined flight path established by the operator based on the vegetation survey grids to be covered, the survey altitude needed for a specific resolution, and wind conditions at the time of the flight using Trimble Access Aerial Imaging V2.0.00.40 (Trimble, Sunnyvale, CA). Using the UX5’s built in GPS system, a flightlog recorded georeferenced images with 80% forward and horizontal overlap. Still images were collected in true color (3 visible bands: Red Blue Green) and were automatically taken with a Sony NEX-5R 16.1 MP camera (Sony Corporation of America, New York, NY) along flight paths. Relevant camera settings for all flights were as follows: no flash, exposure time 1/4000, automatic white balance, and automatic ISO. Pictures were taken by automatic trigger approximately once every second while on flight tracks and were saved in JPEG format to an onboard 16GB SD card. Once the flight area had been covered, the UX5 returned to a pre-defined landing zone and belly landed. Imagery and flight logs were downloaded following completion of individual flights. All flights were done on 14 July 2016 between the hours of 0900 and 1200. Study plots were surveyed at 75, 100, and 120 m above ground level (AGL), resulting in a ground sampling distance (linear distance between center points of adjacent pixels) of 2.4, 3.2 and 3.8 cm, respectively.

We were also interested in any differences in wind conditions during flight operations which could affect aircraft stability and thus image quality. Therefore, we examined weather data which was collected throughout the field season by a consumer-level AcuRite weather station (Chaney Instrument Co, WI). Windspeed measurements were recorded every 12 minutes (default settings), along with the peak windspeed during the 12 minute window.

Raw images were stitched together using Pix4Dmapper Pro (Pix4D, Switzerland, V3.3) to create high resolution mosaics of study plots, which were loaded into ArcGIS 10.6 (ESRI, Redlands, CA) for image classification, and the areas of interest were clipped out. Mosaics were separately classified into 30 class types using an unsupervised classification approach [47] and classes were manually inspected and reclassified into barren, non-shrub, or shrub categories. We selected to employ unsupervised classifications based on preliminary accuracy results when compared to both supervised and random forest classifiers during data exploration. This approach also allowed us to test a simple classification method that requires relatively little technical training and is useful for ecologists with access to a widely used program. Study plots were classified separately to account for any variation in light conditions between plots or any natural variation in land cover type reflectance across the study area. High resolution imagery is often associated with a “salt-and-pepper” effect, where individual pixels are incorrectly classified as different from their majority neighbors [48]. To account for this effect, post processing was done using methods in Chabot and Bird (2013) [49]. This was done by applying a majority filter and boundary clean tools, followed by the removal of patches <0.25m^2^, which were replaced based on the values of nearest neighbours.

We calculated proportion cover as the proportion area represented by each class in relation to the total area surveyed across all plots from classified images for the three flight altitudes. We assessed classification accuracy by generating 100 randomly stratified points within each plot, where the number of points generated for each class is proportional to the relative area occupied by each class. This was repeated for each survey altitude (100 points in each plot, 500 total for each altitude). Standard convention for accuracy assessments is to use georeferenced ground-truth data as the comparative standard for site-specific accuracy assessments, but this can present a problem for very high resolution imagery. Commonly used consumer grade GPS units can vary by several meters in their horizontal accuracy [50, 51], which could result in biased accuracy assessments in heterogeneous land cover habitats. Survey grade GPS units would overcome this problem, but these are financially costly for researchers and were unavailable for this project. Further, each point of ground data collected in this study was not georeferenced and thus unable to be used for creation of an accuracy assessment confusion matrix.

To assess our imagery accuracy in a manner similar to that of commercially purchased imagery, the true classification for assessment points was assessed via visual inspection (manual photointerpretation) of each altitude’s respective high resolution RGB mosaics, which allows relatively clear identification of land cover type for each point. Similar practices with high resolution drone imagery have previously been reported in the literature [49, 52, 53]. It should be noted that visual inspection of imagery is not likely to be 100% accurate, but given the high resolution nature of the imagery, we have a high degree of confidence in correct vegetation class identification. We calculated overall accuracy and kappa coefficients for each flight altitude.

### Statistical analysis

To compare estimates between ground-based linear transects and drone imagery, we examined proportional cover data within cells. Proportional data from ground-based linear transects within cells was obtained by taking the number of data points (steps) for each type (barren, non-shrub, and shrub) and dividing by the total number of data points in each cell. Drone proportional data was produced with two approaches. First, we extracted the proportion of each land cover class within each cell as the number of pixels for each class type divided by the total number of pixels for each respective cell. While this is a common approach to land cover assessments from remotely sensed imagery, any differences between estimates from this method and the field-based transects may simply reflect differences in sampling technique (i.e. assessing the entire cell using the drone vs sampling a small proportion on the ground). To address this discrepancy, we also replicated ground-based data collection by overlaying approximately the same ground-based linear transects within cells in the classified drone imagery. We extracted classification values every meter along the two drone transects within each cell and calculated proportion land cover class for each cell using the number of data points for each class type divided by the total number of data points within each cell.

We calculated Pearson’s correlation coefficients in R v3.4.3 [54] comparing the three methods of data acquisition (ground transects, drone transects, and drone pixel counts) for each cover type. Each method has its own value for a cover type within an individual cell and data are measured on the same scale; therefore deviation from a 1:1 relationship should represent a difference in measurement between methods.

We then used a modified version of the generalized linear mixed model presented in Peterson at al. (2013) [44] to estimate the proportion of land cover type (barren, non-shrub, and shrub) across our five study plots. Models were constructed using PROC GLIMMIX in SAS Studio 3.7 (Cary, NC). We modeled proportional land cover assuming a beta distribution for data constrained between 0 and 1 [55, 56]. To accommodate cells with values of 0 or 1, we transformed data according to Smithson and Verkuilen (2006) [57],

$$y^{\prime }=\frac{y\times (n-1)+0.5}{n}$$

where *n* is equal to the number of data points collected for each method within each cell (i.e. the number of transect points or pixels within a classified cell), *y* is the original proportion cover estimate for each cell, and *y*′ is the adjusted value. By doing so 0’s or 1’s are respectively modified by the gain or loss of one-half the detection limit for each cell. We used a logit link function and a variance components covariance structure. Since we were first interested in the estimates between different drone survey altitudes, we constructed separate models for each drone method (drone pixel counts vs drone transects). These models were produced for each cover type, and only examined the fixed effect of *altitude* (3 levels: 75 m, 100 m, and 120 m AGL). We then constructed another set of models examining the difference between ground estimates, and those from our highest accuracy drone survey altitude. These models included only the single fixed effect of *method* (three levels: ground based transects, drone based transects, and drone pixels counts). For all models we included the random effect of *cell_id* (n = 92). Model fit was assessed via Generalized Chi-Square/DF as a measure of dispersion, and we generated Conditional Pearson’s and Studentized residual plots for each model.

### Ethics statement

Data collection in the area was authorized by Wapusk National Park permit WAP-2015-18846 and WAP-2016-21419. Drone operations were permitted by Transport Canada Special Flight Operations Certificate (File: 5802-11-302, ATS: 15-16-00058646, RDIMS: 11717338). Additionally, the UND Unmanned Aircraft System Research Compliance Committee reviewed and approved project protocols for human privacy and data management (April 10, 2015).

## Results

In July 2016 ground-based assessments were completed by surveying 184 transects in 92 cells, taking approximately 72 researcher-hours. To survey the same plots, drone surveys took 61 min at 75 m AGL (2 flights), 28 min at 100 m AGL (1 flight), and 26 min at 120 m AGL (1 flight). While drone surveys were initially quicker than the ground based field work, post-flight image processing (data management, mosaic creation, image classification, etc.) took approximately 50 hours. Wind conditions during drone flights were mostly similar. The 75 m flights had a mean windspeed of 5.86 km h^-1^ (SD = 1.22, peak speed = 8.70), the 100 m flight had a mean of 5.19 km h^-1^ (SD = 0.72, peak speed = 8.08), and the 120 m flight had a mean of 8.08 km h^-1^ (SD = 1.78, peak speed = 10.56).

### Drone image classification

Unsupervised classifications in ArcGIS produced similar proportion cover results for each altitude based on total enumeration of pixels across the study area, and there were minor differences in overall accuracy and kappa coefficients (Table 1). Notably, the mean accuracy and kappa coefficients decreased with increasing drone survey altitude, but the range of values for both measures overlapped between the three altitudes (Table 1). The lowest altitude surveys at 75m AGL produced the highest mean ± SD overall accuracy of 92.0±0.019%, followed by 90.8±0.036% at 100m AGL, and 88.8±0.024% at 120m AGL.

**Table 1:** Proportion land cover type classification of drone (Trimble UX5) imagery at three altitudes. Proportion values are obtained from the enumeration of pixel types for each land cover class across all 5 plots. Accuracy and kappa statistics presented as mean ± SD, along with the range of values.

	Drone Survey Altitude		
	75m	100m	120m
**Proportion Barren**	0.755	0.761	0.741
**Proportion Non-Shrubs**	0.035	0.032	0.050
**Proportion Shrubs**	0.210	0.207	0.208
**Overall Accuracy**	92.0±0.019% Range: 0.90–0.95%	90.8±0.036% Range: 0.86–0.94%	88.8±0.024% Range: 0.86–0.92%
**Kappa Coefficient**	0.81±0.088 Range: 0.66–0.90	0.79±0.075 Range: 0.70–0.88	0.73±0.113 Range: 0.54–0.83

**Fig 2:**
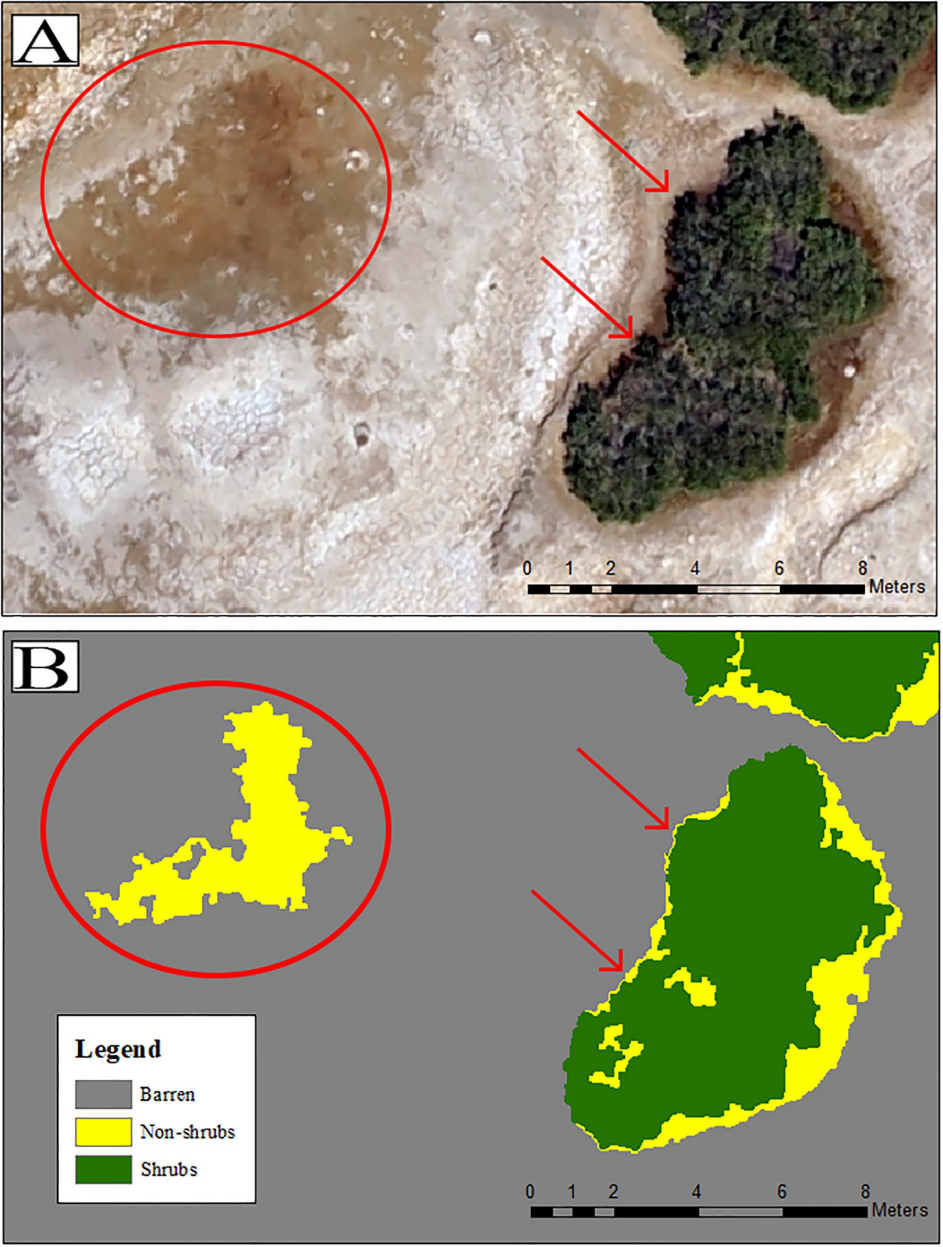
Comparisons between RGB drone imagery and classified product. (A) Example RGB imagery at 120 m AGL (B) final classified image. Post processing tools failed to eliminate the patch of darker barren surface and incorrectly classified the patch as non-shrub vegetation (indicated by the red circle). Shadows along the edge of the vegetation patch were improperly classified as shrubs (indicated by red arrows).

Visual inspection of the RGB mosaics and classified images revealed several consistent errors remaining despite post processing efforts (Fig 2). Distinctions between relatively darker mats of graminoid vegetation (non-shrubs such as *Puccinelia sp*., *Rannunculus sp*.) and darker soils proved difficult for the pixel based classifiers as indicated by higher errors of omission and commission at all altitudes (S1 Appendix). Further, larger shadows from rocks and vegetation were often classified as shrub patches (Fig 2), although smaller shadows were often successfully eliminated via post-processing tools.

### Ground vs drone cover estimates

**Fig 3:**
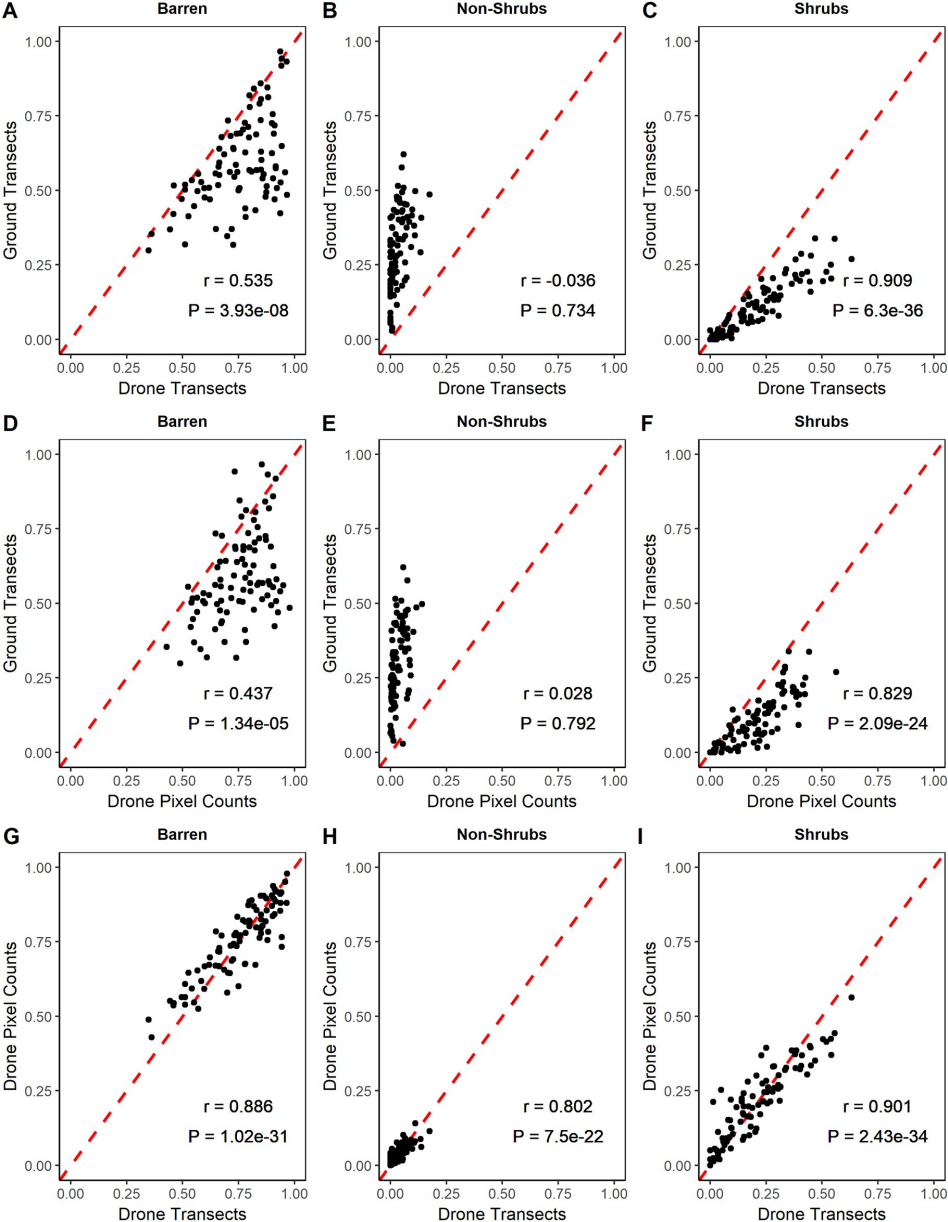
Plotted proportional values and Pearson’s correlation coefficients between three methods of data acquisition (ground transects, drone transects and drone pixel counts). Data presented for each cover type (barren, non-shrubs, and shrubs). Each point represents proportional cover data collected within the same cell (n = 92) for each method. Drone imagery collected at 75 m AGL. Red dashed line represents 1:1 relationship.

We chose to examine correlations using drone estimates from the 75 m AGL flight, which had the highest mean overall accuracy (Table 1). Generally barren and shrub cover types had higher agreement among the three methods of measurement (Fig 3). Non-shrub cover was poorly measured by both drone methods when compared to ground transects (Pearson’s r = -0.036 for drone transects, and r = 0.028 for drone pixel counts), indicating the drone RGB imagery is inadequate for detecting the inconspicuous graminoid and forb species that dominate the non-shrub category. However, both drone methods had high agreement in measurements for all three classes (Fig 3G and 3I).

**Fig 4:**
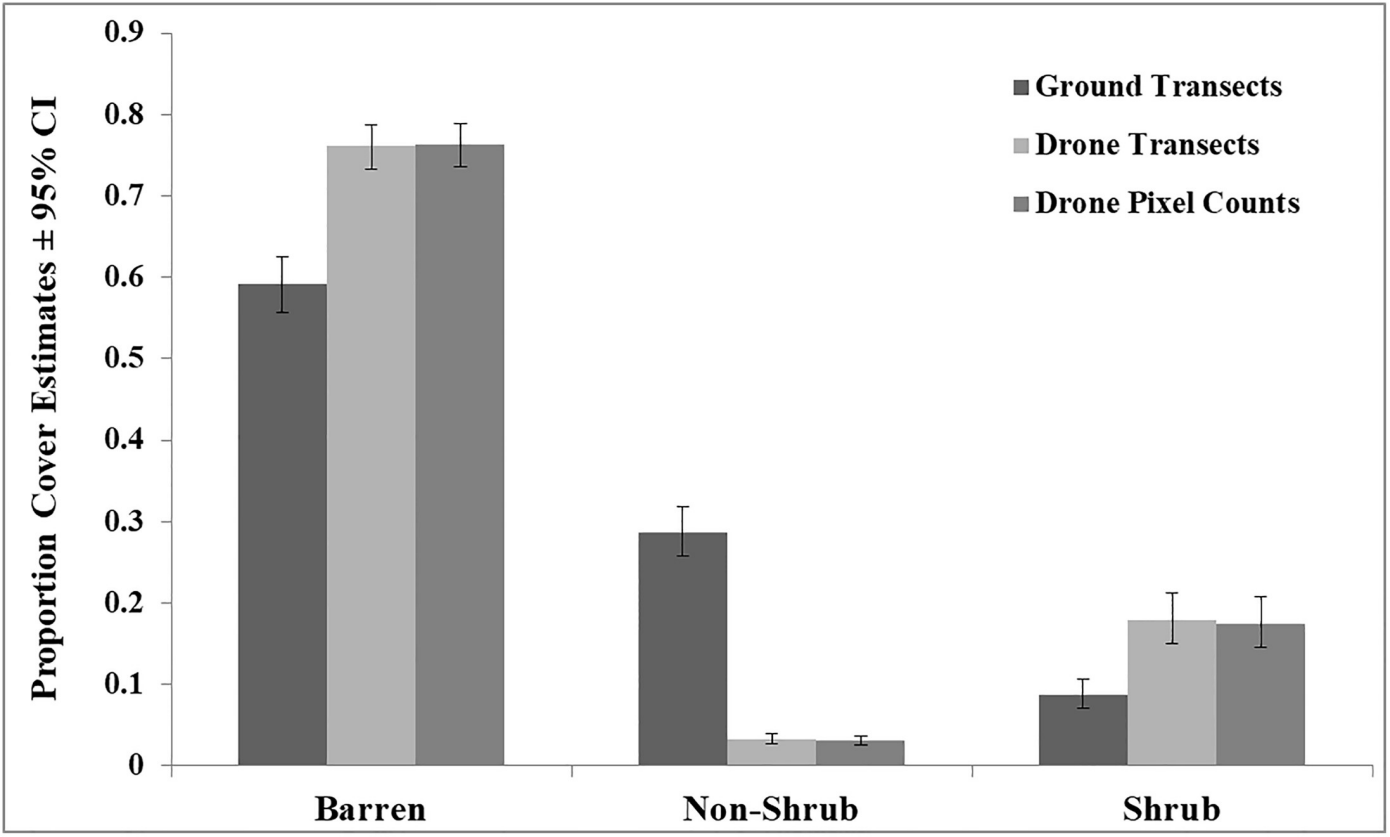
Mixed model estimates of proportion land cover type from three different methods of data collection. Ground transects data collected as linear transects, drone transects are the same transects overlaid on classified drone imagery (see Methods section) with land cover values were extracted every meter, and drone pixel counts based on the enumeration of pixels for each land cover type as a proportion of all pixels in each cell. Drone estimates made from imagery collected at 75 m AGL. Cover data obtained from 92 cells across 5 study plots.

Drone pixel count models indicated significant differences in measurements for barren (F_2,182_ = 16.24, P<0.0001) and non-shrubs (F_2,182_ = 18.56, P<0.0001), but not for shrubs (F_2,182_ = 3.02, P = 0.051) (Table 2). Similarly, drone transects also indicated significant differences in measurements for barren (F_2,182_ = 10.17, P<0.0001) and non-shrubs (F_2,182_ = 10.49, P<0.0001), but not for shrubs (F_2,182_ = 1.30, P = 0.275) (Table 3). Our third set of models examining differences between ground and drone methods indicated that drone methods overestimated barren and shrub categories, but underestimated non-shrubs (Table 4). We plotted model estimates of proportion land cover from only the 75m drone survey in comparison to ground estimates (Fig 4). Mixed model estimates from all three methods indicate higher proportion cover of barren area when compared to shrubs and non-shrub cover (Fig 4). Models showed no evidence of over- or underdispersion. Inspection of residual plots revealed no clear violation of model assumptions.

**Table 2:** Coefficient estimates from Beta GLMM for each cover type (barren, non-shrub, and shrub) as measured by drone pixel counts at altitudes of 75, 100 and 120m AGL. Estimates obtained from 92 observations (cells) across 5 different study plots.

	Cover Type		
	Barren	Non-Shrub	Shrub
*Coefficient Estimate ± SE*			
Intercept	1.274 ± 0.088	-3.418 ± 0.089	-1.590 ± 0.116
100 m AGL*	0.036 ± 0.020	-0.087 ± 0.095	-0.022 ± 0.010
120 m AGL*	-0.075 ± 0.020	0.395 ± 0.086	-0.020 ± 0.010
*Covariance Parameter Estimates ± SE*			
Cell	0.687 ± 0.105	0.320 ± 0.070	1.232 ± 0.191
*Fixed Effect Tests*			
Altitude	F_2,182_ = 16.24, P<0.0001	F_2,182_ = 18.56, P<0.0001	F_2,182_ = 3.02, P = 0.051
*Fit Statistics*			
Generalized Chi-Square/DF	1.00	1.00	1.00

*Baseline comparisons are to measurements made from drone pixel counts at 75 m AGL

**Table 3:** Coefficient estimates from Beta GLMM for each cover type (barren, non-shrub, and shrub) as measured by drone transects at altitudes of 75, 100 and 120m AGL. Estimates obtained from 92 observations (cells) across 5 different study plots.

	Cover Type		
	Barren	Non-Shrub	Shrub
*Coefficient Estimate ± SE*			
Intercept	1.295 ± 0.101	-3.337 ± 0.098	-1.635 ± 0.129
100 m AGL*	0.032 ± 0.033	-0.083 ± 0.113	-0.016 ± 0.025
120 m AGL*	-0.109 ± 0.033	0.355 ± 0.103	0.024 ± 0.025
*Covariance Parameter Estimates ± SE*			
Cell	0.873 ± 0.135	0.307 ± 0.074	1.484 ± 0.231
*Fixed Effect Tests*			
Altitude	F_2,182_ = 10.17, P<0.0001	F_2,182_ = 10.49, P<0.0001	F_2,182_ = 1.30, P = 0.275
*Fit Statistics*			
Generalized Chi-Square/DF	1.00	1.00	1.00

*Baseline comparisons are to measurements made from drone transects at 75 m AGL

**Table 4:** Coefficient estimates from Beta GLMM for each cover type (barren, non-shrub, and shrub) as measured by ground based transects, drone based transects and drone pixel counts at 75 m AGL. Estimates obtained from 92 observations (cells) across 5 different study plots.

	Cover Type		
	Barren	Non-Shrub	Shrub
*Coefficient Estimate ± SE*			
Intercept	0.371 ± 0.072	-0.910 ± 0.074	-2.3488 ± 0.111
Drone Pixel Counts*	0.802 ± 0.066	-2.549 ± 0.082	0.798 ± 0.051
Drone Based Transects*	0.787 ± 0.066	-2.483 ± 0.080	0.825 ± 0.050
*Covariance Parameter Estimates ± SE*			
Cell	0.299 ± 0.056	0.406 ± 0.075	0.969 ± 0.161
*Fixed Effect Tests*			
Method	F_2,182_ = 100.03, P<0.0001	F_2,182_ = 843.77, P<0.0001	F_2,182_ = 160.16, P<0.0001
*Fit Statistics*			
Generalized Chi-Square/DF	1.00	1.00	1.00

*Baseline comparisons are to measurements made from ground based transects

## Discussion

Here we show that by using a fixed-wing drone we were able to survey our study area much faster than ground-based methods, but these savings came at the cost of increased time spent during image processing and classification steps. Cruzan et al. (2016) had similar findings on time management and importantly noted that increases in imagery resolution will require concordant investment in computer processing time and power [25]. Indeed, Fraser et al. (2016) reported drone imagery processing times of up to 10 days when producing ultradense point clouds from highly overlapping imagery [58]. As such, longer flight durations to survey larger areas and ultimately process larger amounts of data may present a limit on the scalability of drone technology in ecological research. Fortunately, the efficiency and time savings gained during the data collection step are likely more relevant to researchers in polar regions where ecological field studies are often limited by shorter operational field seasons [28].

Our simple unsupervised classification approach with RGB imagery was moderately successful when compared to ground-based methods. Overall accuracy assessment and kappa coefficients of the RGB mosaics were relatively high with little difference between altitudes and were quantitatively similar to previous vegetation assessments with fixed-wing drones [27, 59, 60]. We expected accuracy of classifications to increase with lower altitude surveys (higher image resolution), which was supported by our findings (see Table 1). Accuracy and kappa coefficients appeared to increase with higher resolution imagery, but we considered these differences between altitudes to be minimal as the difference between the highest and lowest resolution’s mean overall accuracy was only 3.2%. This likely reflects the minimal difference in ground sampling distances between each altitude, and we suspect that advantages gained by higher resolution RGB imagery were simply not realized by our simple classification approach. Consequently, if similar methods were to be used in the future, we encourage higher altitude drone flights which are more efficient at surveying larger study areas [61]. It is worth noting that our highest survey altitude was the highest allowed under our drone operation permit, and higher altitude flights would require additional permitting.

It is important to consider that differences in environmental conditions between flights could have played a role in image quality and subsequent classifications. Our measurements of wind speed were examined post-hoc study design, and in the future, more fine scale environmental measurements should be collected to formally account for differences among flight operations (i.e. every minute). Although our coarse data indicated slightly higher wind speeds during the 120 m flight, we considered these differences to be minimal and likely played little role in differential image quality between flights. Time-of-day has been shown to be an important consideration for drone image acquisition, due to the differential presence of shadows throughout the day [35]. Although we did not measure cloud cover during this study, our period of flight operations used for classifications all occurred within a three hour window, so changes in light conditions likely did not play a large role in image quality differences between flights. Considerations for light conditions will be important in future drone studies, and researchers may benefit from obtaining images on overcast days to minimize the presence of shadows. This, however, will require high quality sensors to compensate for reduced light conditions [35, 58].

We caution that although we were confident in our visual inspection of RGB imagery for each land cover class, it is possible that accuracy was artificially inflated due to researcher biases. As such, results should be interpreted with care. The lack of georeferenced ground-truthed data in this study represents an obstacle for the future of long-range drone surveys in ecology. If BVLOS surveys become routine in ecology, researchers will not always be present at field sites to validate imagery collected by drones. Therefore, efforts should be made to test aircraft capable of BVLOS flights on smaller scales where comparisons between traditional and drone methods for ecological parameters of interest are conducted, as we did in this study.

Although our model results appeared to overestimate barren and shrub cover while underestimating non-shrubs, similar findings have been reported in the literature [60, 62]. Similar spectral signatures of shrubs and non-shrub species likely played a large role in our misclassifications, which lends support to the apparent need for additional layers of input data (hyperspectral, textural, etc.) to achieve fine-scale classifications [63]. While we recognize that our drone imagery was inadequate at capturing inconspicuous graminoid and forb species (see Fig 3), the use of bare ground coverage has been shown to be a reliable metric for measuring snow goose habitat degradation [64]. As such, our simple drone imagery should be reliable at determining the impact of snow geese on Arctic vegetation communities at a coarse scale. Further, our high altitude drone estimates corroborate the findings of Fraser et al. (2016), who found drone imagery to be a useful method for measuring Arctic shrub communities by combining spectral and structure-from-motion data inputs into their classifiers with an overall accuracy of 82% [58]. These findings support the notion that simple RGB imagery from drones may be more effective for identifying broad scale patterns of conspicuous features, but delineation between more inconspicuous species remains a challenge. Despite post-processing efforts undertaken in ArcGIS, Chabot et al. (2013) suggests that incorporating texture information could help differentiate between classes of land cover with similar spectral properties [49]. More sophisticated techniques such as object based image analysis or random forest classifiers have been used for vegetation assessments from drone imagery and may yield more accurate results, but will come at the cost of increased processing time and requires proficiency in more advanced image analysis techniques [48, 59, 63].

Our estimates of land cover from all three methods generally agree with most recent habitat assessments in the La Pérouse Bay region and that the majority of study plots remain dominated by barren ground, likely as a result of hypersaline conditions [7, 44]. Experimental evidence indicates that in the absence of goose foraging and presence of suitable soil conditions, degraded habitats may recover their graminoid assemblages [65]. While there is some evidence of re-vegetation in long term goose exclosures in supratidal marsh areas at La Pérouse Bay (Rockwell unpublished data 2008–2018), widespread vegetation re-establishment is not yet apparent when compared to historical assessments [43]. Our classifications were restricted to three broad classes of land cover, keeping in-line with previous assessments in the region that used a similar approach [7, 44]. It is possible that increasing the number of classes in our study may produce different accuracy statistics, but consistency in classification types allows us to attempt integration of novel drone technology into long-term ground based datasets. Further, initial inspections of RGB mosaics revealed difficulties in differentiating between several distinct shrub species (e.g. *B*. *glandulosa*, *S*. *planifolia*, *S*. *candida*), indicating that coarse classifications may be more successful. While we did not attempt to distinguish between different species of shrubs, graminoids or forbs in this study and were not the primary objective of this study, the development of drone models and sensors may still play an important role in understanding the impacts of snow geese within their ecosystem, with respect to changing plant communities. Logistic and financial constraints can often prevent repeat surveys by researchers on the ground, but drone flights are easily repeatable and may assist in future monitoring protocols [66]. Ground based approaches may also have their own associated biases such as researcher fatigue or experience level in identifying plants. Drones may help overcome the fatigue bias due to the ability to archive data and spread data collection (image interpretation) over several shorter sessions. In plant community studies where higher spatial coverage is often required for landscape-level inferences, fixed-wing drones may be more advantageous than quadcopter models [25, 27]. If one of the goals of snow goose monitoring involves repeat surveys of vegetation communities, drones may prove a useful tool for quickly surveying larger areas to collect coarse landscape level data. However, ground-based fieldwork will likely still be required if fine-scale data is desired.

Here we have detailed the application of a fixed-wing drone using RGB imagery and a relatively simple classification method for evaluation of snow goose habitat damage. Applications of similar methods have played an important role in understanding polar vegetation [28, 58, 67] but may also be used to research other types of habitat degradation and landscape changes. Potential applications might include changes in salinity, overgrazing, beetle infestations of forests, land-use conversions, and changes in ephemeral wetland coverage. Although we used a simple technique here, future studies could explore the use of more sophisticated multispectral sensors in drones, which have previously been used in fine-scale plant ecology studies [68–70]. Multispectral sensors in drones have been heavily employed in precision agriculture for applications such as measuring the Leaf Area Index in vineyards [71] and estimating nitrogen status in sunflowers (*Helianthus annuus*) [72], while miniaturized hyperspectral sensors have been used for detecting water stress in plants [73] and estimating plant biomass [74]. These sensor types offer unique insights into aspects of plant ecology beyond measuring abundance and distribution, potentially allowing researchers to address a wide variety of ecology phenomenon using drones. The natural progression of these technologies from industry applications to academic research is assisted by decreasing costs and accessibility of miniaturized sensors [75]. However such specialized sensors generally require field calibrations, which may necessitate further expenditures and validation experiments in the field [76]. Any such experiments should consider paired survey designs (see Ahmed et al. 2017) that explicitly compare performance between competing sensors and aircraft design to better facilitate comparisons [70].

The implementation of drones for ecological research in polar regions will ultimately depend on the specifics and scale of the scientific questions being asked. Current government and technological limitations prevent drone use at broad spatial scales, and several studies have noted limitations of current drone based research due to within line-of-sight flight regulations [23, 30, 34, 40]. However if the operation of long-range drone models is eventually outsourced to commercial operations, these regulations may be more easily overcome by industry partners with aircraft regulation expertise. To better facilitate the development of drones for ecological research, we recommend researchers report specifics of their aircrafts as seen in Zweig et al. [77] and Vermeulen et al. [78]. The benefit of this reporting will better inform researchers considering drones as methods for research and monitoring projects in the future.

## Supporting information

S1 Appendix S1 Appendix provides details on study plots, classification scheme used for ground-based linear transects, and confusion matrices from accuracy assessments of drone imagery. (DOCX)

S1 Data S1 Data proportional data for each 50m^2^ cell (n = 92) within each study plot (n = 5). Data summarized as proportion for each method (ground-based linear transects, drone transects, drone pixel count). (XLSX)

## References

[1] Alisauskas RT, Rockwell RF, Dufour KW, Cooch EG, Zimmerman G, Drake KL, et al Harvest, survival, and abundance of midcontinent lesser snow geese relative to population reduction efforts. Wildlife Monographs. 2011;179(1):1–42.

[2] Ankney CD. An embarrassment of riches: too many geese. J Wildl Manage. 1996:217–23.

[3] Jefferies R, Rockwell R, Abraham K. Agricultural Food Subsidies, Migratory Connectivity and Large-Scale Disturbance in Arctic Coastal Systems: A Case Study1. Integrative and Comparative Biology. 2004;44(2):130–9. 10.1093/icb/44.2.130 21680493

[4] Abraham KF, Jefferies RL, Alisauskas RT. The dynamics of landscape change and snow geese in mid‐continent North America. Glob Chang Biol. 2005;11(6):841–55.

[5] Jefferies R, Rockwell R, Abraham K. The embarrassment of riches: agricultural food subsidies, high goose numbers, and loss of Arctic wetlands a continuing saga. Environmental Reviews. 2004;11(4):193–232.

[6] Flemming SA, Calvert A, Nol E, Smith PA. Do hyperabundant Arctic-nesting geese pose a problem for sympatric species? Environmental Reviews. 2016;24(4):393–402.

[7] Rockwell RF, Witte CR, Jefferies R, Weatherhead PJ. Response of nesting savannah sparrows to 25 years of habitat change in a snow goose colony. Ecoscience. 2003;10(1):33–7.

[8] Peterson SL, Rockwell RF, Witte CR, Koons DN. Legacy effects of habitat degradation by Lesser Snow Geese on nesting Savannah Sparrows. The Condor. 2014;116(4):527–37.

[9] Samelius G, Alisauskas RT. Habitat alteration by geese at a large arctic goose colony: consequences for lemmings and voles. Can J Zool. 2009;87(1):95–101.

[10] Milakovic B, Carleton T, Jefferies RL. Changes in midge (Diptera: Chironomidae) populations of sub-arctic supratidal vernal ponds in response to goose foraging. Ecoscience. 2001;8(1):58–67.

[11] Milakovic B, Jefferies R. The effects of goose herbivory and loss of vegetation on ground beetle and spider assemblages in an Arctic supratidal marsh. Ecoscience. 2003;10(1):57–65.

[12] Burgess RM, Ritchie RJ, Person BT, Suydam RS, Shook JE, Prichard AK, et al Rapid growth of a nesting colony of lesser snow geese (Chen caerulescens caerulescens) on the Ikpikpuk River delta, North Slope, Alaska, USA. Waterbirds. 2017;40(1):11–23.

[13] Abraham K, Leafloor J, Lumsden H. Establishment and growth of the lesser snow goose, Chen caerulescens caerulescens, nesting colony on Akimiski Island, James Bay, Northwest Territories. Canadian Field-Naturalist. 1999;113(2):245–50.

[14] Alisauskas RT, Charlwood JW, Kellett DK. Vegetation correlates of the history and density of nesting by Ross’s Geese and Lesser Snow Geese at Karrak Lake, Nunavut. Arctic. 2006:201–10.

[15] Abraham KF. Goose foraging in Arctic habitats with a protocol for a rapid ground based assessment of its impacts on northern plant communities. Prepared for Canadian Wildlife Service, Praire and Northern Region, Winnipeg, MB. 2014.

[16] Jano AP, Jefferies RL, Rockwell RF. The detection of vegetational change by multitemporal analysis of LANDSAT data: the effects of goose foraging. Journal of Ecology. 1998;86(1):93–9.

[17] Jefferies RL, Jano AP, Abraham KF. A biotic agent promotes large‐scale catastrophic change in the coastal marshes of Hudson Bay. Journal of Ecology. 2006;94(1):234–42.

[18] Hogrefe KR, Patil VP, Ruthrauff DR, Meixell BW, Budde ME, Hupp JW, et al Normalized Difference Vegetation Index as an Estimator for Abundance and Quality of Avian Herbivore Forage in Arctic Alaska. Remote Sens. 2017;9(12):1234.

[19] LaRue MA, Stapleton S. Estimating the abundance of polar bears on Wrangel Island during late summer using high-resolution satellite imagery: a pilot study. Polar Biol. 2018;41(12):2621–6.

[20] LaRue MA, Stapleton S, Porter C, Atkinson S, Atwood T, Dyck M, et al Testing methods for using high‐resolution satellite imagery to monitor polar bear abundance and distribution. Wild Soc Bull. 2015;39(4):772–9.

[21] Loarie SR, Joppa LN, Pimm SL. Satellites miss environmental priorities. Trends Ecol Evol. 2007;22(12):630–2. 10.1016/j.tree.2007.08.018 17996978

[22] Chapman A. It’s okay to call them drones. J Unman Veh Syst. 2014;2(02):iii–v.

[23] Anderson K, Gaston KJ. Lightweight unmanned aerial vehicles will revolutionize spatial ecology. Frontiers in Ecology and the Environment. 2013;11(3):138–46. 10.1890/120150

[24] Chabot D, Bird DM. Wildlife research and management methods in the 21st century: Where do unmanned aircraft fit in? J Unman Veh Syst. 2015;3(4):137–55.

[25] Cruzan MB, Weinstein BG, Grasty MR, Kohrn BF, Hendrickson EC, Arredondo TM, et al Small unmanned aerial vehicles (micro-UAVs, drones) in plant ecology. Applications in plant sciences. 2016;4(9):1600041. 10.3732/apps.1600041 PMC5033362 27672518

[26] Chabot D, Carignan V, Bird DM. Measuring habitat quality for least bitterns in a created wetland with use of a small unmanned aircraft. Wetlands. 2014;34(3):527–33.

[27] Marcaccio JV, Markle CE, Chow-Fraser P. Use of fixed-wing and multi-rotor unmanned aerial vehicles to map dynamic changes in a freshwater marsh. J Unman Veh Syst. 2016;4(3):193–202.

[28] Malenovský Z, Lucieer A, King DH, Turnbull JD, Robinson SA. Unmanned aircraft system advances health mapping of fragile polar vegetation. Methods Ecol Evol. 2017.

[29] Mallory ML, Gilchrist HG, Janssen M, Major HL, Merkel F, Provencher JF, et al Financial costs of conducting science in the Arctic: examples from seabird research. Arct Sci. 2018;4(4):624–33.

[30] Barnas AF, Felege CJ, Rockwell RF, Ellis-Felege SN. A pilot(less) study on the use of an unmanned aircraft system for studying polar bears (*Ursus maritimus*). Polar Biol. 2018:1–8. 10.1007/s00300-018-2270-0

[31] Hanson L, Holmquist-Johnson CL, Cowardin ML. Evaluation of the Raven sUAS to detect and monitor greater sage-grouse leks within the Middle Park population. U.S. Geological Survey Open-File Report 2014–1205, 2014 2331–1258.

[32] Rümmler M-C, Mustafa O, Maercker J, Peter H-U, Esefeld J. Measuring the influence of unmanned aerial vehicles on Adélie penguins. Polar Biol. 2015;39(7):1329–34.

[33] Fortune SM, Koski WR, Higdon JW, Trites AW, Baumgartner MF, Ferguson SH. Evidence of molting and the function of “rock-nosing” behavior in bowhead whales in the eastern Canadian Arctic. PLoS ONE. 2017;12(11):e0186156 10.1371/journal.pone.0186156 29166385 PMC5699794

[34] Christie KS, Gilbert SL, Brown CL, Hatfield M, Hanson L. Unmanned aircraft systems in wildlife research: current and future applications of a transformative technology. Front Ecol Environ. 2016;14(5):241–51.

[35] Patterson C, Koski W, Pace P, McLuckie B, Bird DM. Evaluation of an unmanned aircraft system for detecting surrogate caribou targets in Labrador. J Unman Veh Syst. 2015;4(1):53–69.

[36] Zmarz A, Rodzewicz M, Dąbski M, Karsznia I, Korczak-Abshire M, Chwedorzewska KJ. Application of UAV BVLOS remote sensing data for multi-faceted analysis of Antarctic ecosystem. Remote Sensing of Environment. 2018;217:375–88.

[37] Sykora-Bodie ST, Bezy V, Johnston DW, Newton E, Lohmann KJ. Quantifying nearshore sea turtle densities: applications of unmanned aerial systems for population assessments. Sci Rep. 2017;7(1):17690 10.1038/s41598-017-17719-x 29255157 PMC5735099

[38] Ferguson M, Angliss R, Kennedy A, Lynch B, Willoughby A, Helker V, et al Performance of manned and unmanned aerial surveys to collect visual data and imagery for estimating arctic cetacean density and associated uncertainty. J Unman Veh Syst. 2018;6(3):128–54.

[39] Moreland EE, Cameron MF, Angliss RP, Boveng PL. Evaluation of a ship-based unoccupied aircraft system (UAS) for surveys of spotted and ribbon seals in the Bering Sea pack ice. J Unman Veh Syst. 2015;3(3):114–22.

[40] Hodgson A, Kelly N, Peel D. Unmanned aerial vehicles (UAVs) for surveying marine fauna: a dugong case study. PLoS ONE. 2013;8(11):e79556 10.1371/journal.pone.0079556 24223967 PMC3817127

[41] Koski WR, Gamage G, Davis AR, Mathews T, LeBlanc B, Ferguson SH. Evaluation of UAS for photographic re-identification of bowhead whales, *Balaena mysticetus*. J Unman Veh Syst. 2015;3(1):22–9.

[42] Shilts WW, Aylsworth JM, Kaszycki CA, Klassen RA. Canadian shield Geomorphic Systems of North America. 2: Geological Society of America Boulder, Colorado; 1987 p. 119–61.

[43] Weatherhead PJ. Ecological correlates of monogamy in tundra-breeding savannah sparrows. Auk. 1979:391–401.

[44] Peterson SL, Rockwell RF, Witte CR, Koons DN. The legacy of destructive Snow Goose foraging on supratidal marsh habitat in the Hudson Bay lowlands. Arctic, Antarctic, and Alpine Research. 2013;45(4):575–83.

[45] Evans RA, Love RM. The step-point method of sampling-a practical tool in range research. Rangeland Ecology & Management/Journal of Range Management Archives. 1957;10(5):208–12.

[46] Owensby C. Modified step-point system for botanical conposition and basal cover estimates. Journal of Range Management Archives. 1973;26(4):302–3.

[47] Lillesand T, Kiefer RW, Chipman J. Remote sensing and image interpretation: John Wiley & Sons; 2014.

[48] Feng Q, Liu J, Gong J. UAV remote sensing for urban vegetation mapping using random forest and texture analysis. Remote Sens. 2015;7(1):1074–94.

[49] Chabot D, Bird DM. Small unmanned aircraft: precise and convenient new tools for surveying wetlands. J Unman Veh Syst. 2013;1(01):15–24.

[50] Wing MG, Eklund A, Kellogg LD. Consumer-grade global positioning system (GPS) accuracy and reliability. Journal of forestry. 2005;103(4):169.

[51] Arnold LL, Zandbergen PA. Positional accuracy of the wide area augmentation system in consumer-grade GPS units. Computers & Geosciences. 2011;37(7):883–92.

[52] Su L, Gibeaut J. Using UAS Hyperspatial RGB Imagery for Identifying Beach Zones along the South Texas Coast. Remote Sens. 2017;9(2):159.

[53] Pande-Chhetri R, Abd-Elrahman A, Liu T, Morton J, Wilhelm VL. Object-based classification of wetland vegetation using very high-resolution unmanned air system imagery. European Journal of Remote Sensing. 2017;50(1):564–76.

[54] R Core Team. R: A Language and Environment for Statistical Computing. Vienna: R Foundation for Statistical Computing; https://wwwr-projectorg/. 2017.

[55] Ferrari S, Cribari-Neto F. Beta regression for modelling rates and proportions. Journal of Applied Statistics. 2004;31(7):799–815.

[56] Eskelson BN, Madsen L, Hagar JC, Temesgen H. Estimating riparian understory vegetation cover with beta regression and copula models. Forest Science. 2011;57(3):212–21.

[57] Smithson M, Verkuilen J. A better lemon squeezer? Maximum-likelihood regression with beta-distributed dependent variables. Psychological methods. 2006;11(1):54 10.1037/1082-989X.11.1.54 16594767

[58] Fraser RH, Olthof I, Lantz TC, Schmitt C. UAV photogrammetry for mapping vegetation in the low-Arctic. Arct Sci. 2016;2(3):79–102.

[59] Laliberte AS, Herrick JE, Rango A, Winters C. Acquisition, orthorectification, and object-based classification of unmanned aerial vehicle (UAV) imagery for rangeland monitoring. Photogrammetric Engineering & Remote Sensing. 2010;76(6):661–72.

[60] Mora C, Vieira G, Pina P, Lousada M, Christiansen HH. Land cover classification using high‐resolution aerial photography in adventdalen, svalbard. Geografiska Annaler: Series A, Physical Geography. 2015;97(3):473–88.

[61] Linchant J, Lisein J, Semeki J, Lejeune P, Vermeulen C. Are unmanned aircraft systems (UASs) the future of wildlife monitoring? A review of accomplishments and challenges. Mamm Rev. 2015;45(4):239–52.

[62] Breckenridge RP, Dakins M, Bunting S, Harbour JL, Lee RD. Using unmanned helicopters to assess vegetation cover in sagebrush steppe ecosystems. Rangeland ecology & management. 2012;65(4):362–70.

[63] Turner D, Lucieer A, Malenovský Z, King D, Robinson SA. Assessment of Antarctic moss health from multi-sensor UAS imagery with Random Forest Modelling. International journal of applied earth observation and geoinformation. 2018;68:168–79.

[64] Jefferies RL, Rockwell RF. Foraging geese, vegetation loss and soil degradation in an Arctic salt marsh. Applied vegetation science. 2002;5(1):7–16.

[65] Abraham K, Jefferies R, Alisauskas R, Rockwell R. Northern wetland ecosystems and their response to high densities of lesser snow geese and Ross’s geese. Evaluation of special management measures for midcontinent lesser snow geese and Ross’s geese Arctic Goose Joint Venture Special Publication US Fish and Wildlife Service, Washington, DC and Canadian Wildlife Service, Ottawa, Ontario. 2012:9–45.

[66] Sardà‐Palomera F, Bota G, Padilla N, Brotons L, Sardà F. Unmanned aircraft systems to unravel spatial and temporal factors affecting dynamics of colony formation and nesting success in birds. J Avian Biol. 2017;48(9):1273–80.

[67] Lucieer A, Turner D, King DH, Robinson SA. Using an Unmanned Aerial Vehicle (UAV) to capture micro-topography of Antarctic moss beds. International journal of applied earth observation and geoinformation. 2014;27:53–62.

[68] Strecha C, Fletcher A, Lechner A, Erskine P, Fua P. Developing species specific vegetation maps using multi-spectral hyperspatial imagery from unmanned aerial vehicles. ISPRS Annals of the Photogrammetry, Remote Sensing and Spatial Information Sciences. 2012;3:311–6.

[69] Knoth C, Klein B, Prinz T, Kleinebecker T. Unmanned aerial vehicles as innovative remote sensing platforms for high‐resolution infrared imagery to support restoration monitoring in cut‐over bogs. Applied vegetation science. 2013;16(3):509–17.

[70] Ahmed OS, Shemrock A, Chabot D, Dillon C, Williams G, Wasson R, et al Hierarchical land cover and vegetation classification using multispectral data acquired from an unmanned aerial vehicle. Int J Remote Sens. 2017;38(8–10):2037–52.

[71] Mathews AJ, Jensen JL. Visualizing and quantifying vineyard canopy LAI using an unmanned aerial vehicle (UAV) collected high density structure from motion point cloud. Remote Sens. 2013;5(5):2164–83.

[72] Agüera F, Carvajal F, Pérez M. Measuring sunflower nitrogen status from an unmanned aerial vehicle-based system and an on the ground device. Int Arch Photogramm Remote Sens Spat Inf Sci. 2011;38:33–7.

[73] Zarco-Tejada PJ, González-Dugo V, Berni JA. Fluorescence, temperature and narrow-band indices acquired from a UAV platform for water stress detection using a micro-hyperspectral imager and a thermal camera. Remote Sensing of Environment. 2012;117:322–37.

[74] Pölönen I, Saari H, Kaivosoja J, Honkavaara E, Pesonen L, editors. Hyperspectral imaging based biomass and nitrogen content estimations from light-weight UAV. Remote Sensing for Agriculture, Ecosystems, and Hydrology XV; 2013: International Society for Optics and Photonics.

[75] Berra EF, Gaulton R, Barr S. Commercial off-the-shelf digital cameras on unmanned aerial vehicles for multitemporal monitoring of vegetation reflectance and NDVI. IEEE Trans Geosci Remote Sens. 2017;55(9):4878–86.

[76] Tay JY, Erfmeier A, Kalwij JM. Reaching new heights: can drones replace current methods to study plant population dynamics? Plant Ecology. 2018;219(10):1139–50.

[77] Zweig CL, Burgess MA, Percival HF, Kitchens WM. Use of unmanned aircraft systems to delineate fine-scale wetland vegetation communities. Wetlands. 2015;35(2):303–9.

[78] Vermeulen C, Lejeune P, Lisein J, Sawadogo P, Bouché P. Unmanned aerial survey of elephants. PLoS ONE. 2013;8(2):1–7. 10.1371/journal.pone.0054700 23405088 PMC3566131

[79] ESRI. "Canadian Provinces" [basemap]. Scale Not Given. http://services1.arcgis.com/eZaevkfA0RPFQmA8/arcgis/rest/services/Canada_Provinces/FeatureServer. (April 2018).

