# Supplementary material for: A comparison of drone imagery and ground-based methods for estimating the extent of habitat destruction by lesser snow geese (Anser caerulescens caerulescens) in La Pérouse Bay
Source: PLoS One. 2019 Aug 9;14(8):e0217049. doi: 10.1371/journal.pone.0217049 (PMC6688855; doi:10.1371/journal.pone.0217049)
Supplement: S1 Appendix — (DOCX) [file pone.0217049.s001.docx]

**Appendix S1: Further Details on Study Plots and Vegetation Classification**

**Table S1 Further details on rectangular study plots dimensions and number of cells.**

| **Plot Number** | **Area (ha)** | **Number of Cells** | **Year Originally Established** |
| --- | --- | --- | --- |
| 1 | 7 | 28 | 1979^*^ |
| 2 | 3 | 12 | 1999^+^ |
| 3 | 3 | 12 | 1999^+^ |
| 4 | 5 | 20 | 1999^+^ |
| 5 | 5 | 20 | 1999^+^ |

* See Weatherhead, P.J. 1979. Ecological correlates of monogamy in tundra-breeding savannah sparrows. *Auk*. 96: 391-401

+ See Rockwell, R.F., Witte, C.R., Jefferies, R.L., and Weatherhead, P.J. 2003. Response of nesting savannah sparrows to 25 years of habitat change in a snow goose colony. *Ecoscience*. 10: 33-37

**Table S2 Landcover and vegetation classifications used in ground based linear transects. Cover was collapsed into three categories: Bare, Non-Shrub, and Shrub.**

| **Cover Type** | **Common Name** | **Code** | **Classification Type** | |
| --- | --- | --- | --- | --- |
| Mud/bare ground | Mud/bare ground | A | Barren | 0 |
| Algal mat | Algal mat | A1 | Barren | 0 |
| Pond with water | Pond with water | B1+ | Barren | 0 |
| Pond with no water | Pond with no water | B1- | Barren | 0 |
| Stream with water | Stream with water | B2+ | Barren | 0 |
| Stream with no water | Stream with no water | B2- | Barren | 0 |
| Moss | Moss | C3 | Non-Shrub | 1 |
| *Atriplex sp.* | Saltbush | H | Non-Shrub | 1 |
| *Carex subspathacea* | Hoppner’s sedge | C1 | Non-Shrub | 1 |
| *Puccinnelia phyrganodes* | Alkali grass | C1 | Non-Shrub | 1 |
| *Calamagrostis deschampiodes* | Reedgrass | C2 | Non-Shrub | 1 |
| *Festuca rubra* | Fescue | C2 | Non-Shrub | 1 |
| *Empetrum nigrum* | Black crowberry | C4 | Non-Shrub | 1 |
| Unidentified flowering plant | Flowers | C5 | Non-Shrub | 1 |
| *Carex aquatilis* | Watersedge | D | Non-Shrub | 1 |
| *Eriophorum sp.* | Cottongrass | D | Non-Shrub | 1 |
| *Elymus sp.* | Wildrye | E | Non-Shrub | 1 |
| *Salicornia sp.* | Pickleweed | F | Non-Shrub | 1 |
| *Spergularia sp.* | Sandspurry | F* | Non-Shrub | 1 |
| *Senecio sp.* | Ragwort | G | Non-Shrub | 1 |
| *Ranunculus sp.* | Crowfoot | I | Non-Shrub | 1 |
| *Hippuris vulgaris* | Common mare tail | Hv | Non-Shrub | 1 |
| *Petasites sagittatus* | Arrowleaf | Ps | Non-Shrub | 1 |
| *Triglochin maritima* | Seaside arrowgrass | Tm | Non-Shrub | 1 |
| *Triglochin palustris* | Marsh arrowgrass | Tp | Non-Shrub | 1 |
| *Rumex occidentalis* | Western Dock | Ro | Non-Shrub | 1 |
| *Matricia amigua* | Mayweed | Ma | Non-Shrub | 1 |
| *Salix sp.* | Willow | S | Shrub | 2 |
| *Salix brachycarpa* | Shortfruit willow | Sb | Shrub | 2 |
| *Salix candida* | Sageleaf willow | Sc | Shrub | 2 |
| *Salix planifolia* | Diamondleaf willow | Sp | Shrub | 2 |
| *Salix lanata* | Wolly willow | Sl | Shrub | 2 |
| *Salix reticulata* | Netleaf willow | Sr | Shrub | 2 |
| *Myrica gale* | Sweetgale | Mg | Shrub | 2 |
| *Betula glandulosa* | Dwarf birch | Bg | Shrub | 2 |

**75m AGL Confusion Matrices**

**Table S3 Confusion matrix of unsupervised classification results from drone imagery at 75 meters above ground level for plot 1. Accuracy results based on 100 equally stratified random points. Numbers on the diagonal represent correct classifications.**

|  | Barren | Non-Shrub | Shrub | Total Points Allocated | User’s Accuracy |
| --- | --- | --- | --- | --- | --- |
| Barren | 65 | 4 | 0 | 69 | 0.94 |
| Non-Shrub | 1 | 8 | 0 | 9 | 0.89 |
| Shrub | 0 | 0 | 22 | 22 | 1 |
| Total Points Assigned | 66 | 12 | 22 | 100 | - |
| Producer’s Accuracy | 0.98 | 0.67 | 1 | - | - |
| Overall Accuracy | 95.0% | | | | |
| Kappa Coefficient | 0.897 | | | | |

**Table S4 Confusion matrix of unsupervised classification results from drone imagery at 75 meters above ground level for plot 2. Accuracy results based on 100 equally stratified random points. Numbers on the diagonal represent correct classifications.**

|  | Barren | Non-Shrub | Shrub | Total Points Allocated | User’s Accuracy |
| --- | --- | --- | --- | --- | --- |
| Barren | 58 | 0 | 1 | 59 | 0.98 |
| Non-Shrub | 5 | 5 | 0 | 10 | 0.5 |
| Shrub | 2 | 0 | 29 | 31 | 0.94 |
| Total Points Assigned | 65 | 5 | 30 | 100 | - |
| Producer’s Accuracy | 0.89 | 1 | 0.97 | - | - |
| Overall Accuracy | 92.0% | | | | |
| Kappa Coefficient | 0.846 | | | | |

**Table S5 Confusion matrix of unsupervised classification results from drone imagery at 75 meters above ground level for plot 3. Accuracy results based on 100 equally stratified random points. Numbers on the diagonal represent correct classifications.**

|  | Barren | Non-Shrub | Shrub | Total Points Allocated | User’s Accuracy |
| --- | --- | --- | --- | --- | --- |
| Barren | 64 | 2 | 1 | 67 | 0.96 |
| Non-Shrub | 5 | 4 | 0 | 9 | 0.44 |
| Shrub | 0 | 1 | 23 | 24 | 0.96 |
| Total Points Assigned | 69 | 7 | 24 | 100 | - |
| Producer’s Accuracy | 0.93 | 0.57 | 0.96 | - | - |
| Overall Accuracy | 91.0% | | | | |
| Kappa Coefficient | 0.81 | | | | |

**Table S6 Confusion matrix of unsupervised classification results from drone imagery at 75 meters above ground level for plot 4. Accuracy results based on 100 equally stratified random points. Numbers on the diagonal represent correct classifications.**

|  | Barren | Non-Shrub | Shrub | Total Points Allocated | User’s Accuracy |
| --- | --- | --- | --- | --- | --- |
| Barren | 64 | 0 | 0 | 64 | 1 |
| Non-Shrub | 3 | 6 | 0 | 9 | 0.67 |
| Shrub | 2 | 3 | 22 | 27 | 0.81 |
| Total Points Assigned | 69 | 9 | 22 | 100 | - |
| Producer’s Accuracy | 0.93 | 0.67 | 1 | - | - |
| Overall Accuracy | 92.0% | | | | |
| Kappa Coefficient | 0.837 | | | | |

**Table S7 Confusion matrix of unsupervised classification results from drone imagery at 75 meters above ground level for plot 5. Accuracy results based on 100 equally stratified random points. Numbers on the diagonal represent correct classifications.**

|  | Barren | Non-Shrub | Shrub | Total Points Allocated | User’s Accuracy |
| --- | --- | --- | --- | --- | --- |
| Barren | 79 | 1 | 0 | 80 | 0.99 |
| Non-Shrub | 6 | 2 | 2 | 10 | 0.2 |
| Shrub | 1 | 0 | 9 | 10 | 0.9 |
| Total Points Assigned | 86 | 3 | 11 | 100 | - |
| Producer’s Accuracy | 0.92 | 0.67 | 0.82 | - | - |
| Overall Accuracy | 90.0% | | | | |
| Kappa Coefficient | 0.664 | | | | |

**100m AGL Confusion Matrices**

**Table S8 Confusion matrix of unsupervised classification results from drone imagery at 100 meters above ground level for plot 1. Accuracy results based on 100 equally stratified random points. Numbers on the diagonal represent correct classifications.**

|  | Barren | Non-Shrub | Shrub | Total Points Allocated | User’s Accuracy |
| --- | --- | --- | --- | --- | --- |
| Barren | 66 | 2 | 0 | 68 | 0.97 |
| Non-Shrub | 3 | 7 | 0 | 10 | 0.7 |
| Shrub | 0 | 3 | 19 | 22 | 0.86 |
| Total Points Assigned | 69 | 12 | 19 | 100 | - |
| Producer’s Accuracy | 0.97 | 0.58 | 1 | - | - |
| Overall Accuracy | 92.0% | | | | |
| Kappa Coefficient | 0.832 | | | | |

**Table S9 Confusion matrix of unsupervised classification results from drone imagery at 100 meters above ground level for plot 2. Accuracy results based on 100 equally stratified random points. Numbers on the diagonal represent correct classifications.**

|  | Barren | Non-Shrub | Shrub | Total Points Allocated | User’s Accuracy |
| --- | --- | --- | --- | --- | --- |
| Barren | 60 | 0 | 0 | 60 | 1 |
| Non-Shrub | 5 | 5 | 0 | 10 | 0.5 |
| Shrub | 1 | 0 | 29 | 30 | 0.97 |
| Total Points Assigned | 66 | 5 | 29 | 100 | - |
| Producer’s Accuracy | 0.91 | 1 | 1 | - | - |
| Overall Accuracy | 94.0% | | | | |
| Kappa Coefficient | 0.883 | | | | |

**Table S10 Confusion matrix of unsupervised classification results from drone imagery at 100 meters above ground level for plot 3. Accuracy results based on 100 equally stratified random points. Numbers on the diagonal represent correct classifications.**

|  | Barren | Non-Shrub | Shrub | Total Points Allocated | User’s Accuracy |
| --- | --- | --- | --- | --- | --- |
| Barren | 65 | 1 | 1 | 67 | 0.97 |
| Non-Shrub | 7 | 2 | 1 | 10 | 0.2 |
| Shrub | 2 | 0 | 21 | 23 | 0.91 |
| Total Points Assigned | 74 | 3 | 23 | 100 | - |
| Producer’s Accuracy | 0.87 | 0.67 | 0.91 | - | - |
| Overall Accuracy | 88.0% | | | | |
| Kappa Coefficient | 0.723 | | | | |

**Table S11 Confusion matrix of unsupervised classification results from drone imagery at 100 meters above ground level for plot 4. Accuracy results based on 100 equally stratified random points. Numbers on the diagonal represent correct classifications.**

|  | Barren | Non-Shrub | Shrub | Total Points Allocated | User’s Accuracy |
| --- | --- | --- | --- | --- | --- |
| Barren | 64 | 1 | 2 | 67 | 0.96 |
| Non-Shrub | 5 | 4 | 0 | 9 | 0.44 |
| Shrub | 3 | 3 | 18 | 24 | 0.75 |
| Total Points Assigned | 72 | 8 | 20 | 100 | - |
| Producer’s Accuracy | 0.89 | 0.5 | 0.9 | - | - |
| Overall Accuracy | 86.0% | | | | |
| Kappa Coefficient | 0.697 | | | | |

**Table S12 Confusion matrix of unsupervised classification results from drone imagery at 100 meters above ground level for plot 5. Accuracy results based on 100 equally stratified random points. Numbers on the diagonal represent correct classifications.**

|  | Barren | Non-Shrub | Shrub | Total Points Allocated | User’s Accuracy |
| --- | --- | --- | --- | --- | --- |
| Barren | 79 | 2 | 0 | 81 | 0.97 |
| Non-Shrub | 4 | 6 | 0 | 10 | 0.6 |
| Shrub | 0 | 0 | 9 | 9 | 1 |
| Total Points Assigned | 83 | 8 | 9 | 100 | - |
| Producer’s Accuracy | 0.95 | 0.75 | 1 | - | - |
| Overall Accuracy | 94.0% | | | | |
| Kappa Coefficient | 0.807 | | | | |

**120m AGL Confusion Matrices**

**Table S13 Confusion matrix of unsupervised classification results from drone imagery at 120 meters above ground level for plot 1. Accuracy results based on 100 equally stratified random points. Numbers on the diagonal represent correct classifications.**

|  | Barren | Non-Shrub | Shrub | Total Points Allocated | User’s Accuracy |
| --- | --- | --- | --- | --- | --- |
| Barren | 65 | 2 | 0 | 67 | 0.97 |
| Non-Shrub | 5 | 5 | 0 | 10 | 0.5 |
| Shrub | 0 | 1 | 22 | 23 | 0.96 |
| Total Points Assigned | 70 | 8 | 22 | 100 | - |
| Producer’s Accuracy | 0.93 | 0.63 | 1 | - | - |
| Overall Accuracy | 92.0% | | | | |
| Kappa Coefficient | 0.831 | | | | |

**Table S14 Confusion matrix of unsupervised classification results from drone imagery at 120 meters above ground level for plot 2. Accuracy results based on 100 equally stratified random points. Numbers on the diagonal represent correct classifications.**

|  | Barren | Non-Shrub | Shrub | Total Points Allocated | User’s Accuracy |
| --- | --- | --- | --- | --- | --- |
| Barren | 56 | 3 | 0 | 59 | 0.95 |
| Non-Shrub | 7 | 3 | 0 | 10 | 0.3 |
| Shrub | 2 | 2 | 27 | 31 | 0.87 |
| Total Points Assigned | 65 | 8 | 27 | 100 | - |
| Producer’s Accuracy | 0.86 | 0.38 | 1 | - | - |
| Overall Accuracy | 86.0% | | | | |
| Kappa Coefficient | 0.733 | | | | |

**Table S15 Confusion matrix of unsupervised classification results from drone imagery at 120 meters above ground level for plot 3. Accuracy results based on 100 equally stratified random points. Numbers on the diagonal represent correct classifications.**

|  | Barren | Non-Shrub | Shrub | Total Points Allocated | User’s Accuracy |
| --- | --- | --- | --- | --- | --- |
| Barren | 67 | 1 | 0 | 68 | 0.99 |
| Non-Shrub | 5 | 4 | 0 | 9 | 0.44 |
| Shrub | 2 | 2 | 19 | 23 | 0.83 |
| Total Points Assigned | 74 | 7 | 19 | 100 | - |
| Producer’s Accuracy | 0.91 | 0.57 | 1 | - | - |
| Overall Accuracy | 90.0% | | | | |
| Kappa Coefficient | 0.776 | | | | |

**Table S16 Confusion matrix of unsupervised classification results from drone imagery at 120 meters above ground level for plot 4. Accuracy results based on 100 equally stratified random points. Numbers on the diagonal represent correct classifications.**

|  | Barren | Non-Shrub | Shrub | Total Points Allocated | User’s Accuracy |
| --- | --- | --- | --- | --- | --- |
| Barren | 62 | 4 | 0 | 66 | 0.94 |
| Non-Shrub | 5 | 5 | 0 | 10 | 0.5 |
| Shrub | 0 | 2 | 22 | 24 | 0.92 |
| Total Points Assigned | 67 | 11 | 22 | 100 | - |
| Producer’s Accuracy | 0.93 | 0.45 | 1 | - | - |
| Overall Accuracy | 89.0% | | | | |
| Kappa Coefficient | 0.777 | | | | |

**Table S17 Confusion matrix of unsupervised classification results from drone imagery at 120 meters above ground level for plot 5. Accuracy results based on 100 equally stratified random points. Numbers on the diagonal represent correct classifications.**

|  | Barren | Non-Shrub | Shrub | Total Points Allocated | User’s Accuracy |
| --- | --- | --- | --- | --- | --- |
| Barren | 79 | 2 | 0 | 81 | 0.98 |
| Non-Shrub | 8 | 2 | 0 | 10 | 0.2 |
| Shrub | 0 | 3 | 6 | 9 | 0.67 |
| Total Points Assigned | 87 | 7 | 6 | 100 | - |
| Producer’s Accuracy | 0.91 | 0.29 | 1 | - | - |
| Overall Accuracy | 87.0% | | | | |
| Kappa Coefficient | 0.540 | | | | |
